# Utilization of Nutmeg (*Myristica fragrans* Houtt.) Seed Hydrodistillation Time to Produce Essential Oil Fractions with Varied Compositions and Pharmacological Effects

**DOI:** 10.3390/molecules25030565

**Published:** 2020-01-28

**Authors:** Mohamed A. Ibrahim, Charles L. Cantrell, Ekaterina A. Jeliazkova, Tess Astatkie, Valtcho D. Zheljazkov

**Affiliations:** 1 National Center for Natural Product Research, University of Mississippi, Oxford, MS 38677, USA; mmibrahi@olemiss.edu; 2 U.S. Department of Agriculture, Agricultural Research Service, Natural Products Utilization Research Unit, Oxford, MS 38677, USA; charles.cantrell@usda.gov; 3 Central Oregon Agriculture Research and Extension Center, Oregon State University, 850 NW Dogwood Ln, Madras, OR 97741, USA; Ekaterina.Jeliazkova@oregonstate.edu; 4 Faculty of Agriculture, Dalhousie University, PO Box 550, Truro, NS B2N 5E3, Canada; astatkie@dal.ca; 5 Crop and Soil Science Department, Oregon State University, 3050 SW Campus Way, 109 Crop Science Building, Corvallis, OR 97331, USA

**Keywords:** Regression models, α-phellandrene, 3-carene, *p*-cymene, limonene, α-thujene, α-pinene, camphene, sabinene, β-pinene, myrcene, α-terpinene, γ-terpinene, terpinolene, myristicin

## Abstract

The intent of this study was to utilize distillation timeframes (DT) of nutmeg (*Myristica fragrans*) essential oil (EO) to generate fractions with differential chemical compositions and bioactivity. Ten fractions were captured at the following distillation timeframes: 0.0–0.5, 0.5–1.0, 1.0–2.5, 2.5–5.0, 5.0–10, 10–30, 30–60, 60–90, 90–120, and 120–240 min. In addition, a control EO was collected from a straight 0–240 min non-stop distillation. ANOVA and advanced regression modeling revealed that the produced EO fractions possess substantial variation in the concentration of potentially desired compounds. The concentrations (%) of α-phellandrene, 3-carene, *p*-cymene, limonene, α-thujene, α-pinene, camphene, sabinene, β-pinene, and myrcene decreased, while the concentrations (%) of α-terpinene, γ-terpinene, terpinolene, and myristicin increased in later DT fractions. Nutmeg EO showed some antimalarial activity against *Plasmodium falciparum* D6, but did not exhibit significant antifungal activity. In general, nutmeg seed oil yields increased with an increase of DT. These results may be utilized by industries using nutmeg EO.

## 1. Introduction

*Myristica fragrans* Houtt., (Myristicaceae), a plant species indigenous to Indonesia, is widely distributed in the humid tropical and coastal regions, and is known as “nutmeg”, for being used in the production of the spices nutmeg and mace [1,2,3]. Historically, nutmeg was believed to be imported to Europe by Arab traders during the 12th century [4], where grated nutmeg was used as a sachet, and the Romans used it as incense. In the 1600s nutmeg became a pricy commercial spice in the Western world [5].

The fruit, similar in shape to the apricot, is eaten locally and once mature splits into two parts, exposing a crimson-colored aril, the mace, which is surrounding the brown seed nutmeg [6,7,8]. The nutmeg is the main source for two distinct spices, nutmeg and mace, where the seed kernel inside the fruits is the source of the spice named “nutmeg”, while the dried flesh around the seed is the source of the spice named “mace” [7]. The mace oil is also produced from the bark, leaf, and flower. Nutmeg oil production is about 500–1200 kg per hectare and the oil is characterized by its unique pungent fragrance and warm taste [9]. Nutmeg is listed among the most traded commodities in the world according to the Product Complexity Index (PCI); 81.7% of the nutmeg export market worldwide is mainly coming from 5 countries; India, Indonesia, Netherlands, The United Arab Emirates (UAE), and Sri Lanka, while the rest is coming from other countries. According to the Office of Export Controls (OEC), India is considered to be the leading exporter of nutmeg worldwide where the market has reached $107 million in 2016. It is mainly cultivated in Kerala, Tamil Nadu, Karnataka, and North East India regions. Although India is the largest producer, the produced amount is not enough to cover local consumption. Indonesia is the world’s top producer of nutmeg (~50% of the world’s production) making up 29% of the world’s market share in 2016 ($96.6 million from nutmeg exports). In Europe, Netherlands is the largest importer and exporter of nutmeg (nutmeg exports were worth $27.1 million in 2016 constituting ~8.1% of the global market share), where it mainly imports its nutmeg from Indonesia and re-exports it to other countries such as the United States and European Union countries.

Nutmeg extracts and essential oil (EO) are used in new drug developments in India, China, and other tropical countries due to the various pharmacological activities. Sabinene (20.2%), terpinen-4-ol (12.1%), safrole (10.3%), α-pinene (9.7%), β-phellandrene (6.6%), and γ-terpinene (5.9%) were reported to be some of the main compounds of nutmeg EO, which is extracted by distillation [10,11]. Nutmeg has been used in folk medicine for multiple purposes such as an antimicrobial, antioxidant, psychostimulant, and antithrombotic [12]. In Ayurveda, the seeds had been used to treat poor digestion, insomnia, and urinary incontinence [13]. Currently nutmeg is known for the treatment of various health disorders, such as bowel movement irregularities, rheumatoid arthritis, and muscle spasm [8]. In some countries like Zanzibar, nutmeg was chewed as an alternative to smoking marijuana [14]. Some studies have highlighted the potential of nutmeg as an anticonvulsant, analgesic, and anti-inflammatory herb. Lignans and neolignans [15,16,17] have been reported from the plant with various pharmacological properties including anticancer and antidepressant activities. Nutmeg is also known to control vomiting and relax spasms as well as possessing insecticidal, fungicidal, and bactericidal activities [18]. The nutmeg EO has been used externally due to its anti-inflammatory effect [15]. Most tropical countries recognized nutmeg in their food and medicinal value herbal lists due to its easy collection and its biological activities. The seeds have been taken internally in the treatment of diarrhea, dysentery, vomiting, and abdominal distension, while externally the seed is used to treat toothache and rheumatic pain [19]. According to Zhang et al. [19], nutmeg EO could alleviate the Complete Freund’s Adjuvant-injection (CFA) induced joint swelling, via inhibition of COX-2 expression and blood substance P level, which highlights the potential of nutmeg oil to control chronic pain. Nutmeg EO has shown antibacterial activity against Gram positive and Gram negative bacteria such as *Escherichia coli*, *Salmonella choleraesuis*, and *Staphylococcus aureus* [20]. Based on previous research and reports, nutmeg EO has significant potential for use in the medicinal and fragrance industries. There is lack of sufficient research on nutmeg EO elution as a function of distillation time. Therefore, the hypothesis of this study was that capturing the nutmeg essential oil (EO) in sequential timeframes during hydrodistillation would generate fractions containing unique compositions and bioactivity.

## 2. Results

### 2.1. Effect of Distillation Time (DT) on Oil Profile

**Figure 1:**
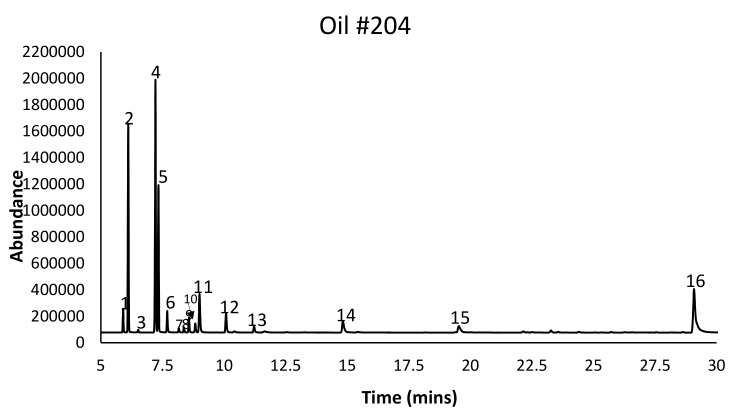
Gas chromatography-flame ionization detector (GC–FID) representative chromatogram from 0 to 240 min distillation time with α-thujene (**1**), α-pinene (**2**), camphene (**3**), sabinene (**4**), β-pinene (**5**), myrcene (**6**), α-phellandrene (**7**), 3-carene (**8**), α-terpinene (**9**), *p*-cymene (**10**), limonene (**11**), γ-terpinene (**12**), terpinolene (**13**), terpinen-4-ol (**14**), safrole (**15**), and myristicin (**16**) indicated.

Ten nutmeg EO fractions were captured at the following distillation timeframes (DT): 0.0–0.5, 0.5–1.0, 1.0–2.5, 2.5–5.0, 5.0–10, 10–30, 30–60, 60–90, 90–120, 120–240 min, and through a non-stop 240 min distillation. The hydrodistillation process employed in this study mimics the process employed by the commercial nutmeg oil extraction facilities. The chemical profiles of these EO fractions were compared to nutmeg EO from the control treatment (non-stop 240 min distillation). DT had significant effects on all of the measured responses, and especially on nutmeg EO profile (Figure 1). Nutmeg EO started coming out almost immediately, and the oil yield was 0.7% in the first half minute (Table 1). Overall, the greatest oil yield among fractions was obtained in the 10–30 min DT. The sum of oil yields from different fractions was approximately equal to the oil yield in the non-stop control, indicating no significant losses in the process of capturing the fractions sequentially.

**Figure 2:**
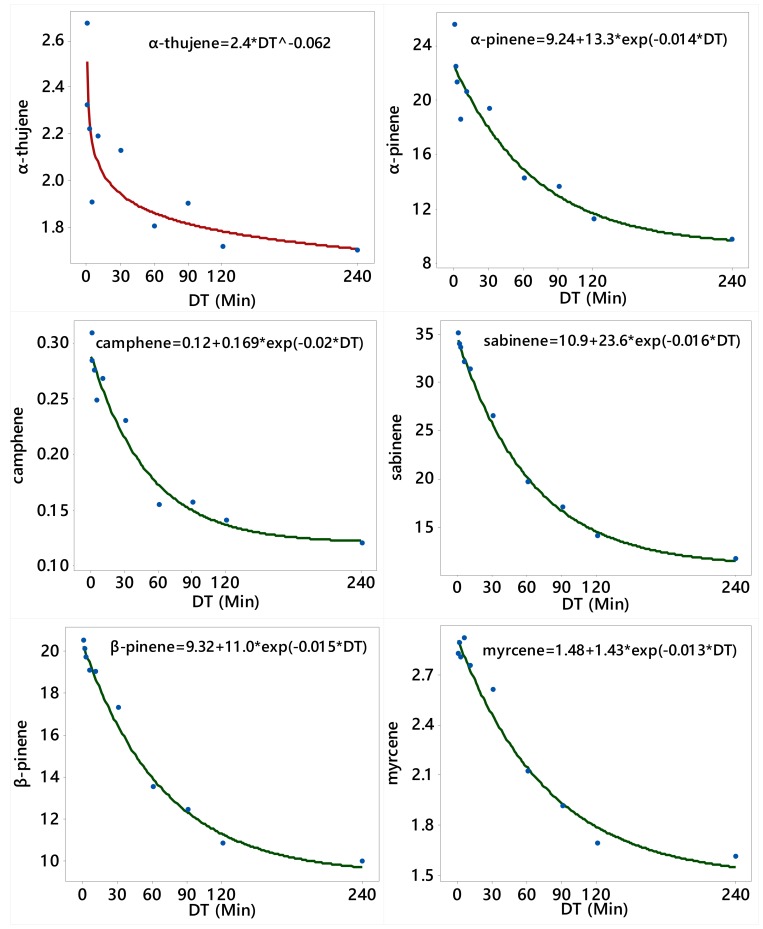
Plot of distillation time (DT) vs. the concentrations (%) of α-thujene, α-pinene, camphene, sabinene, β-pinene, and myrcene along with the fitted (solid line) power regression model (α-thujene) and asymptotic (convex) regression model for the other five constituents.

**Figure 3:**
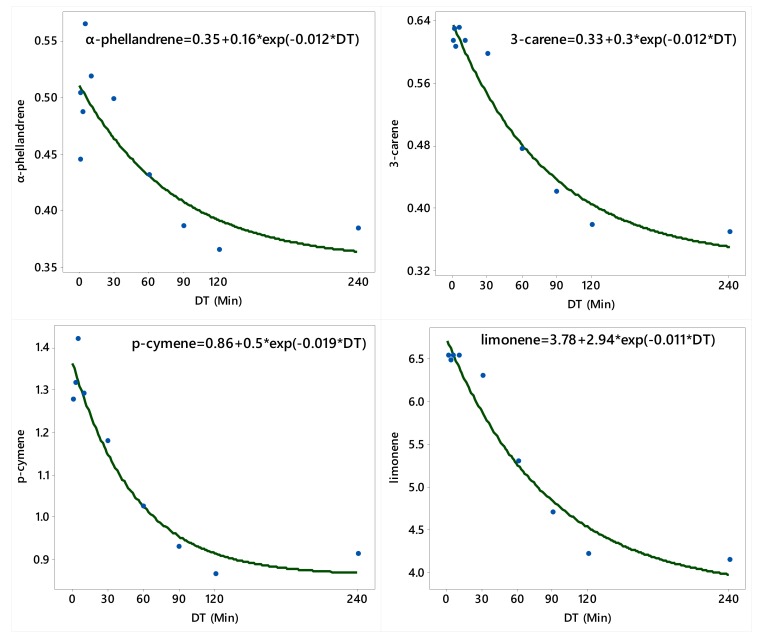
Plot of distillation time (DT) vs. the concentrations (%) of α-phellandrene, 3-carene, *p*-cymene, and limonene along with the fitted (solid line) asymptotic (convex) regression model.

**Figure 4:**
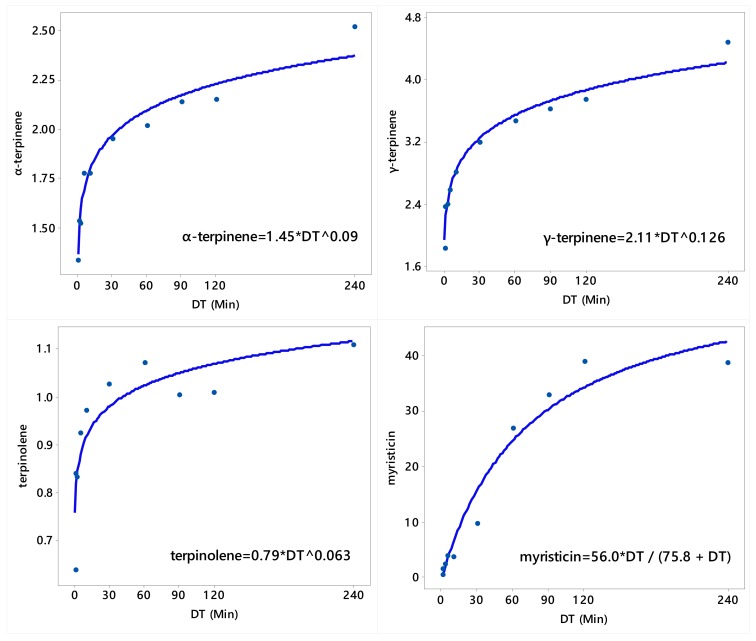
Plot of distillation time (DT) vs. the concentrations (%) of α-terpinene, γ-terpinene, terpinolene, and myristicin along with the fitted (solid line) power regression model (for α-terpinene, γ-terpinene, and terpinolene) and Michaelis–Menten regression model for myristicin.

Generally, the concentrations (%) of α-thujene, α-pinene, camphene, sabinene, β-pinene, α-phellandrene, 3-carene, p-cymene, and limonene in the oil decreased with the increase in the duration of the DT (Table 1 and Table 2, Figure 2 and Figure 3). On the other side, the concentrations (%) of α-terpinene, γ-terpinene, terpinolene, and myristicin increased with the increase in the duration of the DT (Table 3, Figure 4).

Overall, the concentrations of α-thujene α-pinene, camphene, sabinene, and β-pinene were the highest in the earlier fractions (0–0.5 min) and lower in later fractions (90–120 and 120–240 min) (Table 1). The concentrations of myrcene, α-phellandrene, and 3-carene were generally high in the earlier fractions and lower in the later fractions (Table 2). Conversely, the concentration of α-terpinene was low in the initial fractions and gradually increased to max out in the 120–240 min fraction. The concentration of *ρ*-cymene and limonene was low in the initial fraction, and then increased in the middle fractions, followed by decrease in later fractions (Table 2). On the other hand, safrole was the highest in the 30–60 min DT fraction and non-detectable in the 0–0.5 min fraction, while myristicin was the highest in the 60–90, 90–120, and 120–240 min fractions (Table 3).

### 2.2. Effect of Distillation Time (DT) on Antimalarial and Antimicrobial Activities

Nutmeg seeds EO obtained at various time frames were tested for their antimalarial activity against *Plasmodium falciparum D6*, where nutmeg EO at 120–240 min DT was found to be weakly active followed by nutmeg EO at 10–30 min DT, and the control nutmeg EO (nonstop 0–240 min DT), respectively (Table 4). However, nutmeg EO at 1–2.5 min DT was found to be the most active against *Plasmodium falciparum D6*. The nutmeg EO fraction collected at 120–240 min DT was found to be slightly active against *C. neoformans;* however, no significant activity was observed against *C. albicans*, *A. fumigates*, *E. coli*, MRSA, *P. aeruginosa*, *K. pneumonia* and VRE (Table 4). These activity screens represent primary screening data and the activity levels were not active enough to justify further dose-response evaluations.

## 3. Discussion

Many reports have highlighted the importance of nutmeg EO and the use of hydrodistillation in generating nutmeg EO, however in-depth study was needed for evaluating the captured nutmeg EO in sequential timeframes during hydrodistillation in terms of their chemical compositions and bioactivity. The concentration of low boiling point compounds generally was high in the initial DT fractions, while the concentration of compounds with higher boiling points was higher in the later fractions.

The concentration of three major oil constituents (α-pinene BP 156 °C, sabinene 163–165 °C, and β-pinene BP 166 °C) varied significantly as a function of the DT, instead of their boiling points. Although it may seem logical that the concentrations of the EO constituents would vary as a function of their boiling points, there are many additional factors that have a greater influence on the final concentration and yield [13]. These differences in concentrations of the EO constituents unrelated to their comparable boiling points could be attributed to the variation in the availability degree of various tissues for extraction. The oil from 0–0.5 min DT had the highest concentration (%) of α-pinene, β-pinene, and sabinene compared to the oils from the other DT.

Comparing the antimicrobial activities of the four representative fractions (EO at DT 1–2.5 min, EO at DT 10–30 min, EO at DT 120–240 min, and EO at DT 0–240 min) revealed slight potency of EO at DT 120–240 min against *C. neoformans* (IC_50_ 195.206 μg/mL) compared to the other three fractions (IC_50_ >200 μg/mL). Considering the variation of the EO (%) highlight the presence of myristicin in EO at DT 120–240 min, that agrees with what is known about the activity of myristicin against *Cryptococcus neoformans* [21]. Similarly, comparing the antimalarial activities of the four representative fractions revealed that EO at 1–2.5 min DT has higher inhibitory effect towards *P. falciparum* compared to the other three fractions. The variation of the EO (%) highlight the presence of sabinene in EO at 1–2.5 min DT, which agrees with what is known about the activity of sabinene against *P. falciparum* [22].

## 4. Materials and Methods

### 4.1. Hydrodistillation and the Collection of Nutmeg EO Fractions During Different Timeframes (DT)

The nutmeg (*Myristica fragrans*) seed oil was extracted via hydrodistillation of whole seed using 2-L hydrodistillation units (Heartmagic, Rancho Santa Fe, CA, USA) as described previously [23]. The experiment was conducted at Oregon State University in Corvallis, Oregon, while the essential oil analyses were performed at the USDA-ARS, Natural Product Utilization Research Unit located at the University of Mississippi in Oxford (Mississippi, MS, USA). The certified bulk nutmeg seed used in this study originated from Indonesia, and was purchased from StarWest Botanicals (Sacramento, CA, USA).

All DT were performed in 3 replicates and the EO fractions were captured in a sequential order. The nutmeg EOs were collected in glass vials, separated from water, measured on an analytical scale, and kept in a freezer until the gas chromatography analyses could be performed. Nutmeg EO yield (content) was expressed as grams EO per 100 g of nutmeg seeds.

### 4.2. Procedure for Preparing the Nutmeg for Hydrodistillation

One hundred grams of nutmeg seeds were quartered by hand using a knife, then blended in 500 mL water for 1 min in a blender at high speed. The generated material mix was then transferred into the 2 L hydrodistillation flask, where an additional 500 mL of water was used to wash the sides of the blender and added to the 2 L hydrodistillation flask as well. The nutmeg seeds were blended in water in order to eliminate essential oil losses, as reported previously [13,23].

### 4.3. Gas Chromatography, Mass Spectroscopy, Flame Ionization Detector (GC/MS/FID) Analysis

Using a micropipette, 50 μL of nutmeg oil from each sample was transferred into a 10 mL volumetric flask. Samples were brought to volume with CHCl_3_. A 1 mL aliquot of each diluted nutmeg EO sample was placed by glass pipet into a GC vial for analysis.

Nutmeg EO samples were analyzed by GC–MS–FID on an Agilent (Santa Clara, CA, USA) 7890A GC system equipped with an Agilent 5975C inert XL MSD with triple axis detector and an Agilent 7693 autosampler (Santa Clara, CA, USA). Chemical standards and oils were analyzed using a DB-5 column (30 m × 0.25 mm fused silica capillary column, film thickness of 0.25 µm) operated using the following conditions: injector T 240 °C; column temp., 60 to 240 °C at 3 °C/min, held at 240 °C for 5 min; carrier gas, He; injection volume, 1 µL (split ratio 25:1); MS mass range from 50 to 550 *m*/*z*; filament delay of 3.5 min; injection volume, 1 μL (split ratio 50:1); FID temperature was 300 °C. Post-column splitting was performed so that 50% of outlet flow proceeds to FID and 50% to mass spectrometry (MS) detection.

Compounds were identified by Retention Index and Kovat Index analysis [24], direct comparison of MS and retention time to authentic standards, and/or comparison of mass spectra with those reported in the National Institute of Standards and Technology (NIST) mass spectra database. Commercial standards were purchased for all but one (α-thujene) constituents analyzed to provide unequivocal identification. Commercial standards of α-pinene, camphene, sabinene, β-pinene, α-phellandrene, 3-carene, α-terpinene, *p*-cymene, limonene, γ-terpinene, terpinolene, terpinen-4-ol, safrole, and myristicin were obtained from Sigma–Aldrich (St. Louis, MO, USA), and myrcene, from Fluka (Buchs, Switzerlands). Standards were injected and compared with retention time and mass spectra data of oil and used for identification.

Compounds were quantified by performing area percentage calculations based on the total combined FID area. For example, the area for each reported peak was divided by total integrated area from the FID chromatogram from all reported peaks and multiplied by 100 to arrive at a percentage. The percentage of a peak is a percentage relative to all other constituents integrated in the FID chromatogram.

### 4.4. Statistical Analyses

One-way analysis of variance was conducted to determine the effect of distillation time (DT: 0.0–0.5, 0.5–1.0, 1.0–2.5, 2.5–5.0, 5.0–10, 10–30, 30–60, 60–90, 90–120, and 120–240 min as well as straight 0-240 min (Control)) on nutmeg EO content (%), and the concentration (%) of α-thujene, α-pinene, camphene, sabinene, β-pinene, myrcene, α-phellandrene, 3-carene, α-terpinene, p-cymene, limonene, γ-terpinene, terpinolene, terpinen-4-ol, safrole, and myristicin. For each response, the validity of model assumptions was verified by examining the residuals as described in Montgomery [25]. Since the effect of distillation time was significant (*p*-value < 0.05) on all response variables, multiple means comparison was completed using Tukey’s studentized range test (HSD) at the 5% level of significance, and letter groupings were generated. This multiple means comparison method was used because of the low experimental error, and the method’s ability to control Type I experimentwise (family) error rate, which is very important when there are such a large number (11) of means to compare. The analysis was completed using the GLM (General Linear Model) Procedure of SAS (Statistical Analysis System) software [26].

The relationships between distillation time (DT; the 10 timeframes excluding the control) and the concentrations of α-pinene, camphene, sabinene, β-pinene, myrcene, α-phellandrene, 3-carene, *p*-cymene, and limonene were adequately described by the asymptotic (convex) model (Equation (1)); the relationships between DT and the concentrations of α-thujene, α-terpinene, γ-terpinene, and terpinolene were adequately described by the power model (Equation (2)); and the relationship between DT and the concentration (%) of myristicin was adequately described by Michaelis–Menten model (Equation (3)). There was no definite relationship between DT and nutmeg EO content, as well as between DT and the concentrations of terpinen-4-ol and safrole. Since all these three models shown in Equation (1), Equation (2), and Equation (3) are nonlinear, their parameters were estimated iteratively using the NLIN Procedure of SAS [26] and the fitted models met all model adequacy requirements described in Bates and Watts [27].

(1) $$Y=\theta_{1}-\theta_{2}e^{-\theta_{3}x}+\varepsilon$$

(2) $$Y=\theta_{1}X^{\theta_{2}}+\varepsilon$$

(3) $$Y=\frac{\theta_{1}x}{\theta_{2}+x}+\varepsilon$$

where Y is the dependent (response) variable, X is the independent (DT) variable, and ε is the error term assumed to have normal distribution with constant variance. Validity of the normality, constant variance, and independence assumptions on the error terms were verified by examining the residuals [27].

### 4.5. Antimalarial Activity Testing of Nutmeg EO from Various DT

The antimalarial activity of the nutmeg seeds EOs fractions from different treatments (all in two replicates) was tested using a method described previously [28] at the National Center for Natural Product Research (NCNPR), The University of Mississippi, University, MS, USA.

### 4.6. Antimicrobial Activity Testing

The antimicrobial testing of the nutmeg seeds EOs was also performed at the NCNPR, University, MS. The primary screening for antimicrobial activity of nutmeg seeds EOs obtained from the DT treatments (all in two replicates) were tested for antifungal activity against *Candida albicans*, *C. glabrata*, *C. krusei*, *Aspergillus fumigatus*, *Cryptococcus neoformans* and antibacterial potential against Gram positive bacteria *Staphylococus aureus*, methicillin resistant *S. aureus* and *Mycobacterium intracellulare* and Gram negative bacteria *Escherichia coli* and *Pseudomonas aerogenosa* at a concentration of 50 μg/mL. The % inhibition was calculated following the method published previously [28]. The antifungal activity was tested using amphotericin B, the antibacterial drug control was ciprofloxacin.

## 5. Conclusions

This study highlights the potential to produce nutmeg EO fractions with diverse compositions and bioactivity from the same set of seeds. Capturing EO at sequential timeframes can generate EO fractions containing desired characteristics that best suites the needed industrial applications. For example, nutmeg EO with higher concentrations of α-thujene, α-pinene, camphehe, sabinene, and β-pinene can be obtained if it is captured during the first minutes of the distillation. The regression models developed in this study can be utilized to predict EO yield and composition of fractions at any given duration of DT. The regression models could also be useful to compare data from reports on oil yield and composition.

## Figures and Tables

**Table 1:** Mean essential oil (EO) content (%) and concentration (%) of α-thujene, α-pinene, camphene, sabinene, and β-pinene obtained from ten hydrodistillation timeframes (DT) and control (straight 240 min).

DT (min)	EO Content	α-Thujene	α-Pinene	Camphene	Sabinene	β-Pinene
0.0–0.5	0.684 c	2.67 a	25.5 a	0.309 a	35.0 a	20.5 a
0.5–1.0	0.258 de	2.32 ab	22.4 ab	0.284 ab	33.9 ab	20.1 a
1.0–2.5	0.382 cde	2.22 abc	21.2 ab	0.275 ab	33.5 ab	19.7 ab
2.5–5.0	0.421 cde	1.91 bc	18.5 bc	0.248 b	32.0 abc	19.1 abc
5.0–10	0.742 c	2.19 abc	20.5 b	0.268 ab	31.3 abc	19.0 abc
10–30	1.405 b	2.13 abc	19.3 b	0.230 b	26.5 bc	17.3 bc
30–60	0.593 cd	1.80 bc	14.2 cd	0.154 c	19.6 de	13.5 d
60–90	0.430 cde	1.90 bc	13.6 d	0.157 c	17.0 ef	12.4 de
90–120	0.209 e	1.71 c	11.2 d	0.141 c	14.1 fg	10.8 e
120–240	0.518 cde	1.70 c	9.8 d	0.120 c	11.7 g	9.9 e
0–240 Control	5.811 a	2.30 ab	19.7 b	0.241 b	25.6 cd	16.7 c

Within each column, means sharing the same letter are not significantly different.

**Table 2:** Mean concentration (%) of myrcene, α-phellandrene, 3-carene, α-terpinene, *p*-cymene, and limonene obtained from ten hydrodistillation timeframes (DT) and control (straight 240 min).

DT (min)	Myrcene	α-Phellandrene	3-Carene	α-Terpinene	*p*-Cymene	Limonene
0.0–0.5	2.82 ab	0.444 bcd	0.613 a	1.33 e	1.060 bcd	5.72 cd
0.5–1.0	2.89 a	0.504 abc	0.629 a	1.53 de	1.276 ab	6.53 a
1.0–2.5	2.80 ab	0.486 abc	0.606 a	1.52 de	1.315 ab	6.48 a
2.5–5.0	2.91 a	0.564 a	0.630 a	1.77 cd	1.419 a	6.53 a
5.0–10	2.75 ab	0.518 ab	0.613 a	1.78 cd	1.291 ab	6.53 a
10–30	2.61 ab	0.498 abc	0.596 a	1.95 bc	1.179 abcd	6.29 ab
30–60	2.12 c	0.431 bcd	0.475 ab	2.01 bc	1.024 bcd	5.29 d
60–90	1.91 cd	0.386 cd	0.421 ab	2.13 b	0.929 cd	4.70 e
90–120	1.68 d	0.365 d	0.377 b	2.15 b	0.864 d	4.21 ef
120–240	1.61 d	0.384 cd	0.369 b	2.52 a	0.912 d	4.14 f
0–240 Control	2.50 b	0.451 abcd	0.540 ab	1.92 bc	1.227 abc	5.84 bc

Within each column, means sharing the same letter are not significantly different.

**Table 3:** Mean concentration (%) of γ-terpinene, terpinolene, terpinen-4-ol, safrole, and myristicin obtained from ten hydrodistillation timeframes (DT) and control (straight 240 min).

DT (min)	γ-Terpinene	Terpinolene	Terpinen-4-ol	Safrole	Myristicin
0.0–0.5	1.83 h	0.636 e	1.03 e	0.000 g	0.47 f
0.5–1.0	2.36 g	0.838 cd	2.09 de	0.785 f	1.44 e
1.0–2.5	2.40 g	0.832 d	2.97 cd	1.228 ef	2.39 de
2.5–5.0	2.57 fg	0.924 bcd	4.13 abc	1.714 cde	3.79 d
5.0–10	2.80 efg	0.971 abcd	4.11 abc	1.564 cde	3.71 d
10–30	3.19 cde	1.024 ab	5.13 a	2.283 bc	9.75 c
30–60	3.46 bcd	1.069 ab	4.50 ab	3.273 a	26.94 b
60–90	3.62 bc	1.003 abc	2.90 cd	2.590 ab	32.87 ab
90–120	3.73 b	1.008 ab	1.91 de	2.158 bcd	38.77 a
120–240	4.47 a	1.107 a	1.35 e	1.483 def	38.57 a
0–240 Control	3.07 def	0.938 bcd	3.25 bcd	1.996 bcd	13.16 c

Within each column, means sharing the same letter are not significantly different.

**Table 4:** Antimalarial and antimicrobial activities of selected fractions (percent inhibition).

DT (min)	^a^ *P. falciparum D6*	^b^ *C. neoformans*	^b^ *C. albicans*	^b^ *E. coli*
1.0–2.5	39	0	10	4
10–30	16	9	9	8
120–240	14	55	11	6
0–240	18	0	6	0

No recorded inhibition observed for these tested fractions against *A. fumigates*, methicillin resistant Staphylococcus aureus (MRSA), *P. aeruginosa*, *K. pneumonia,* and vancomycin resistant Enterococci (VRE). ^a^ Test concentration of 16 ug/mL. ^b^ Test concentration of 200 ug/mL.
